# Preparation of Palladium-Supported Periodic Mesoporous Organosilicas and their Use as Catalysts in the Suzuki Cross-Coupling Reaction

**DOI:** 10.3390/ma6041554

**Published:** 2013-04-17

**Authors:** Jorge A. Corral, María I. López, Dolores Esquivel, Manuel Mora, César Jiménez-Sanchidrián, Francisco J. Romero-Salguero

**Affiliations:** 1 Department of Organic Chemistry, Nanochemistry and Fine Chemistry Research Institute (IUIQFN), Faculty of Sciences, University of Córdoba, Campus de Rabanales, Marie Curie Building, Ctra. Nnal. IV, km 396, Córdoba 14071, Spain; E-Mails: jorgeacg82@gmail.com (J.A.C.); marisa_lopez_martinez@hotmail.com (M.I.L.); q82momam@uco.es (M.M.); qo1jisac@uco.es (C.J.-S.); 2 Department of Inorganic and Physical Chemistry, Center for Ordered Materials, Organometallics and Catalysis (COMOC), Ghent University, Krijgslaan 281, Building S3, Ghent 9000, Belgium; E-Mail: maesquiv.EsquivelMerino@UGent.be

**Keywords:** periodic mesoporous organosilica, palladium nanoparticles, hydrogen chemisorption, temperature programmed reduction, Pd-supported catalysts, Suzuki cross-coupling

## Abstract

Three periodic mesoporous materials, *i.e.*, two organosilicas with either ethylene or phenylene bridges and one silica, have been used as supports for Pd nanoparticles. All Pd-supported samples (1.0 wt%) were prepared by the incipient wetness method and subsequently reduced in an H_2_ stream at 200 °C. Both hydrogen chemisorption and temperature programmed reduction experiments revealed significant differences depending on the support. Pd^2+^ species were more reducible on the mesoporous organosilicas than on their silica counterpart. Also, remarkable differences on the particle morphology were observed by transmission electron microscopy. All Pd-supported samples were active in the Suzuki cross-coupling reaction between bromobenzene and phenylboronic acid.

## 1. Introduction

In recent years, periodic mesoporous organosilicas (PMOs) have aroused great interest due to their potential applications in different fields such as adsorption, catalysis and biology, among others [1,2]. Besides high surface areas and narrow pore size distributions in the mesopore range, they have organic bridges homogeneously distributed in their pore walls. Consequently, they are more hydrophobic than their silica counterparts, thus generally improving their performance for such applications [3].

Besides some common features between PMOs and their silica counterparts, the presence of organic groups in the framework of PMOs can be a real advantage for an increased stabilization, dispersion and activation of metal nanoparticles. In an early example, Inagaki *et al.* [4] exploited the hydrophobic character of PMOs to improve the catalytic performance of Au particles in the vapor phase epoxidation of propene to propene oxide using H_2_ and O_2_. They used a Ti-substituted ethylene-bridged PMO as support with a Si/Ti ratio of 48. The conversion and selectivity were 3.9% and *ca*. 97%, respectively, both higher than those achieved with other supports with similar Si/Ti ratios, such as Ti-MCM-41, Ti-MCM-48 and Ti-silicalite. A significant decrease in H_2_ consumption due to the enhanced generation of H_2_O_2_ in the hybrid material was observed. Also, a large pore PMO with ethylene and phenylene bridges was used as support for Au nanoparticles with an average size from 3 to 15 nm [5]. This material was very active in the Ullmann coupling of different iodoarenes to the corresponding biphenyls, with yields above 80% when using K_3_PO_4_ as base in N-methylpyrrolidone. The catalyst was reusable for at least five runs. Interestingly, Au supported in SBA-15, TiO_2_, SiO_2_ or activated carbon showed rather low activity. In order to avoid the aggregation of the metal precursor during the reduction step and thus prevent the formation of large Au particles in the external surface of PMOs, an ethylene-bridged PMO was selectively functionalized with n-octadecyltrimethoxysilane in the external surface and, subsequently, with aminopropyltrimethoxysilane in the mesopore walls prior to the impregnation step [6]. After reduction with hydrogen, Au particles with sizes between 3 and 5 nm were found inside the pore channels, whereas they reached *ca*. 50 nm in a similar PMO without anchored octadecyl groups. The confined Au nanoparticles in the PMO were active in the reduction of methylene blue dye with NaBH_4_.

Pd nanoparticles of *ca*. 1.9 nm were supported on a phenylene-bridged PMO by an impregnation method [7]. A comparison with Pd loaded (in all cases at a Pd content of 6 wt.%) on MCM-41 and MCM-41 with grafted phenyl groups revealed that the Pd/PMO catalyst was more active and selective than the MCM-41 based catalysts in the Ullman coupling of iodobenzene and bromobenzene to biphenyl in water. The higher catalytic performance of Pd/PMO (biphenyl yield > 90%) was ascribed to its higher affinity for toluene and its more hydrophobic surface, thus facilitating the adsorption and diffusion of the reactants. Despite the basic conditions, the catalyst was reusable at least four times. Also, a PMO with alkylimidazolium ionic liquid bridges was utilized to complex Pd(II) species and subsequently used as catalyst for the Suzuki-Miyaura coupling between aryl halides and arylboronic acids [8]. The yields to the corresponding bis-aryl compounds were good for the aryl chlorides and excellent for the aryl bromides. Poisoning experiments revealed that the PMO was only a reservoir for soluble Pd species which were the actual catalyst. The reusability and recovery of the catalyst were good because the PMO framework retains the formed Pd nanoparticles and prevents their agglomeration. A structurally analogous non-functionalized SBA-15 extensively deactivated after the first run, thus pointing out the decisive role of the bridges.

Herein, we report the synthesis of Pd nanoparticles supported on two periodic mesoporous organosilicas with either ethylene (Ethane-PMO) or phenylene (Benzene-PMO) bridges. The supports and the corresponding metallic phases have been characterized using different techniques in order to determine the influence of the support on the Pd-supported nanoparticles. These Pd-supported PMOs have been compared with their silica counterpart (PMS). The Suzuki cross-coupling reaction between bromobenzene and phenylboronic acid, which is of a great interest in organic synthesis due to the formation of a new C-C bond, was used to test the performance of these materials as catalysts [9,10,11,12].

## 2. Results and Discussion

### 2.1. Characterization of the Supports

**Figure 1:**
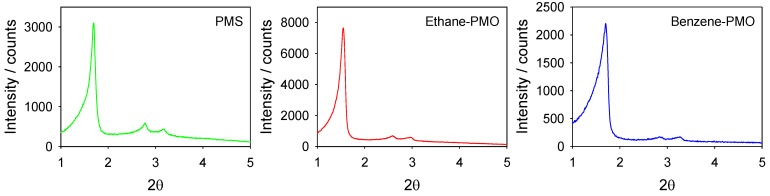
X-ray powder diffraction (XRD) patterns of the mesoporous materials used as supports.

The XRD patterns of all materials exhibited low angle (100) peaks with a *d*-spacing between 52 and 57 Å as well as broad and short second-order (110) and (200) peaks at higher incidence angles indicative of materials with 2D hexagonal (*P*6*mm*) mesopore structures (Figure 1 and Table 1) [13].

**Table 1:** Physicochemical properties of periodic mesoporous materials.

Material	a_0_ (A) ^1^	BET surface area (m^2^ g^−1^)	Pore volume (cm^3^ g^−1^)	Pore diameter (A)	Wall thickness (A) ^2^
PMS	60	965	1.16	44	16
Ethane-PMO	66	1070	1.13	38	28
Benzene-PMO	60	1083	0.73	31	29

^1^ Unit-cell dimension calculated from a_0_ = (2*d*_100_/$\sqrt{3}$); ^2^ Estimated from (a_0_—pore diameter).

**Figure 2:**
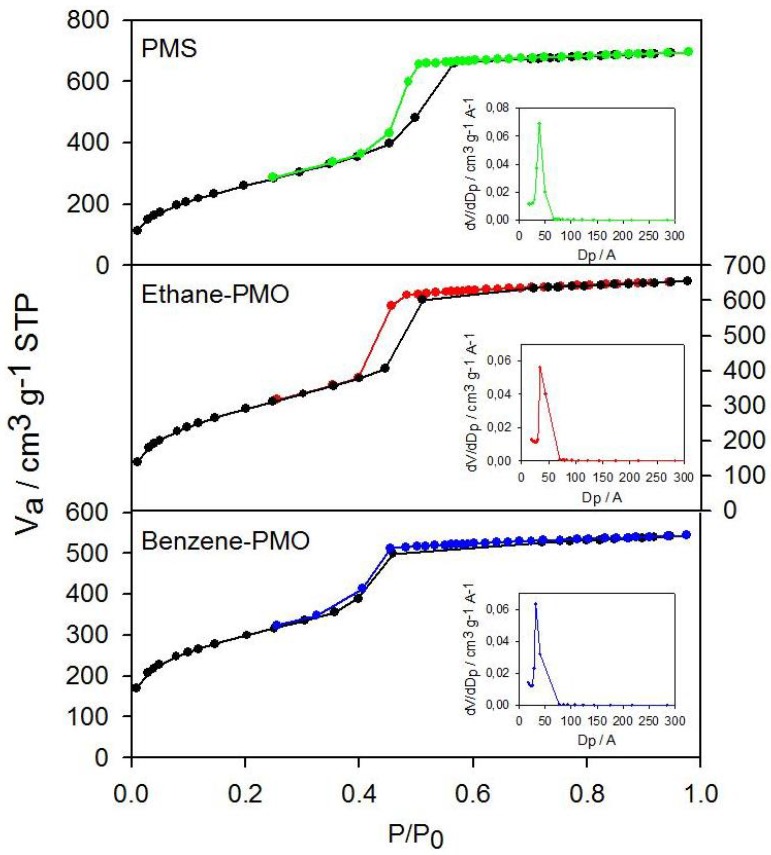
Nitrogen adsorption-desorption isotherms and pore size distribution curves (insets) of the mesoporous materials used as supports.

The N_2_ adsorption–desorption isotherms for all samples were of type IV with the step at a relative pressure of 0.35–0.6 (*i.e.*, typical of mesoporous solids) (Figure 2). All materials exhibited a high surface area of around 1000 m^2^ g^−1^ (Table 1). The average pore diameter and the narrow pore size distribution confirmed the presence of pores in the mesopore range. The pore diameter increased in the following sequence: Benzene-PMO < Ethane-PMO < PMS, as indicated by the shift of the condensation step to higher pressure values. Furthermore, the pore wall thickness was higher for the organosilicas than for their silica counterpart.

### 2.2. Characterization of the Metal Phase

**Figure 3:**
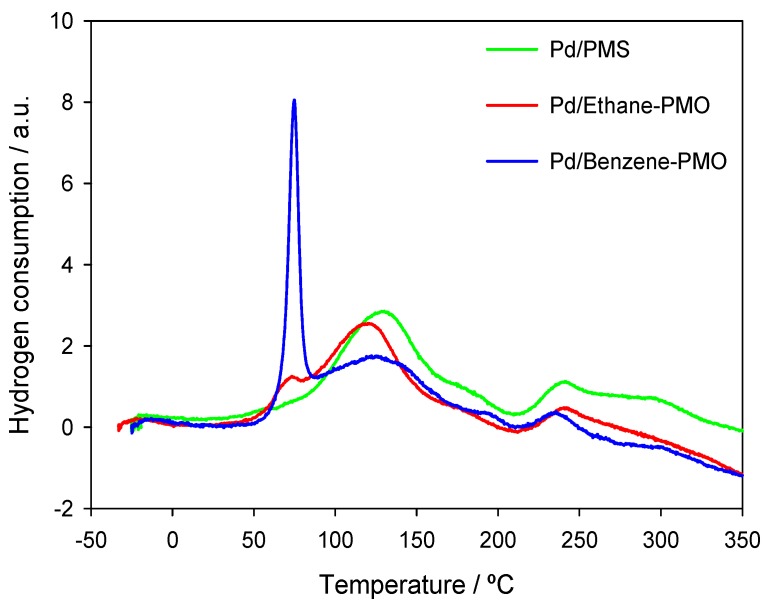
Temperature-programmed reduction (TPR) profiles for the different Pd-supported mesoporous materials.

The metal phase was characterized via TPR and H_2_ chemisorption tests. Figure 3 depicts the TPR curves for the three samples. The TPR curve of sample Pd/PMS was dominated by an intense band centered at *ca.* 130 °C attributable to the reduction of Pd^2+^ to Pd^0^. In addition, some additional small peaks appeared at *ca*. 180, 240 and 295 °C. Material Pd/Ethane-PMO exhibited a similar profile with the main peak at a slightly lower temperature (120 °C), thus involving a weaker interaction of the Pd^2+^ species with the support [14]. Moreover, a small reduction peak was also observed at 75 °C. This peak was the major one in sample Pd/Benzene-PMO. This band could be tentatively assigned to the reduction of Pd^2+^ located in hydrophobic regions, whereas those signals above 80 °C would correspond to Pd^2+^ species coordinated to silanol groups, *i.e.*, those found in hydrophilic regions. The particular behavior of Benzene-PMO as support must be due to the presence of phenylene bridges in its pore walls which favor the adsorption of Pd(acac)_2_ in the hydrophobic regions through the interaction of Pd^2+^ with the π clouds. Similar interactions with other metals in Benzene-PMOs have been previously reported [15]. Also, the formation of π-complexes between Pd^2+^ species and carbon surfaces is well known [16]. Thus, Benzene-PMO as support can be considered to have characteristic of both silica and carbon materials due to the presence of surface silanol groups and benzene rings, respectively, on its surface. Moreover, negative peaks below *ca*. 100 °C due to the decomposition of palladium hydride were not observed in any Pd-supported material studied. Thus, the reduction of the impregnated materials at subambient temperatures to yield Pd nanoparticles able to absorb H_2_ within their structure can be ruled out [17,18].

**Figure 4:**
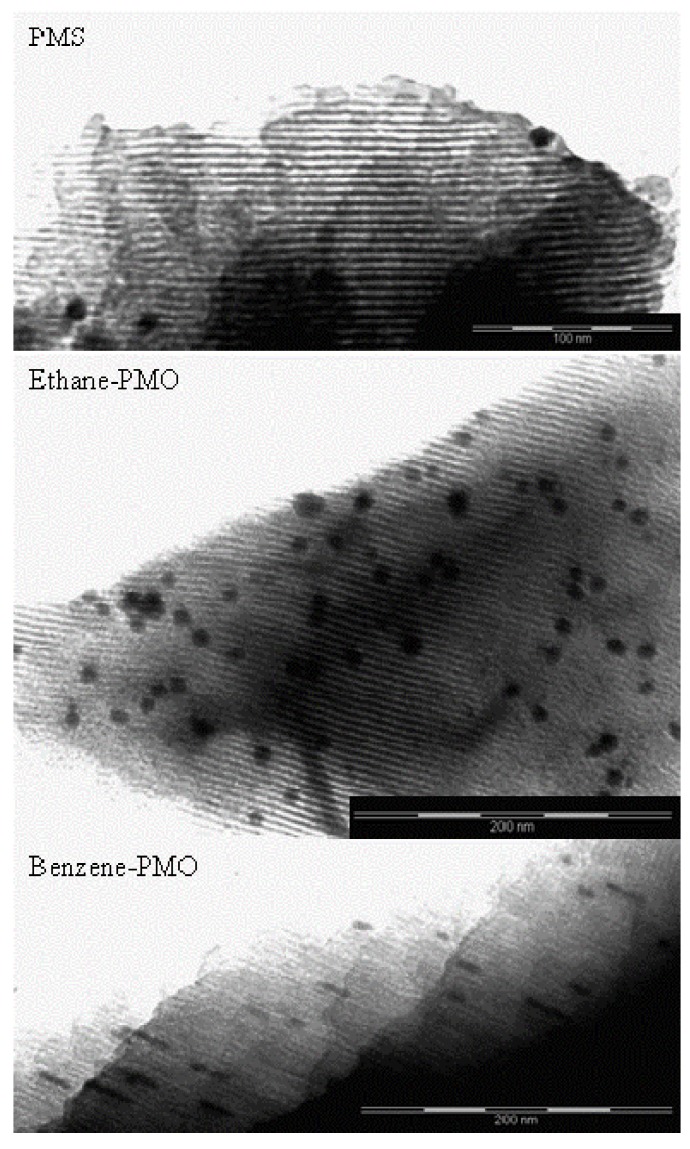
Representative TEM images of Pd-supported samples, showing the pore system of the mesoporous materials and some metal particles.

Transmission electron microscopy (TEM) images of the Pd-supported materials are shown in Figure 4. All of them exhibited the one-dimensional channels characteristic of mesoporous structures. Only a few Pd crystallites with a diameter of *ca.* 10 nm were found on the external surface of sample Pd/PMS, indicating that most Pd nanoparticles were embedded in the pore walls and thus well dispersed in this material. Particles with a size smaller than 1 nm, which may be occluded in the channels, are sometimes difficult to be distinguished from the support with this technique. In contrast, a large number of spherical Pd nanoparticles was visible in sample Pd/Ethane-PMO with diameters ranging from 1.5 to 11 nm. Again, the material Pd/Benzene-PMO was quite peculiar. Unlike the other two supports, the material with phenylene bridges promoted the formation of elongated Pd nanoparticles of about 3 nm wide which were incorporated in the pore channels occupying their whole diameter.

The results of the hydrogen chemisorption experiments are summarized in Table 2. The volume of H_2_ adsorbed on these materials as well as the metal surface area and dispersion depended on the support, increasing in the order Benzene-PMO < Ethane-PMO < PMS. Accordingly, the particle size decreased in the opposite direction, in agreement with the TEM results. The particle size of 3.2 nm determined by H_2_ chemisorption in Pd/PMS pointed out the existence of small Pd particles incorporated in its pore walls. According to the H_2_ chemisorption results and TEM images, this was not the case for Pd/Ethane-PMO, where a considerable fraction of Pd particles seemed to be located on the external surface. Nevertheless, the size of Pd nanoparticles supported on Benzene-PMO calculated by chemisorption was not as reliable as for the other two supports since they were clearly not spherical. In general, the calculated particle diameters were close to those reported for other mesoporous supports [19].

**Table 2:** Metallic properties of Pd nanoparticles supported on mesoporous materials determined by hydrogen chemisorption.

Support	Volume of H_2_ adsorbed (cm^3^ g^−1^)	Metal surface area (m^2^ g_metal_^−1^)	Dispersion (%)	Particle diameter (nm)
PMS	0.370	156	35	3.2
Ethane-PMO	0.229	97	22	5.2
Benzene-PMO	0.211	89	20	5.6

### 2.3. Catalytic Activity

**Scheme 1:**
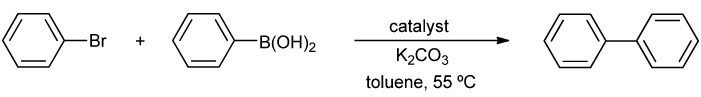
Suzuki cross-coupling reaction between bromobenzene and phenylboronic acid.

**Figure 5:**
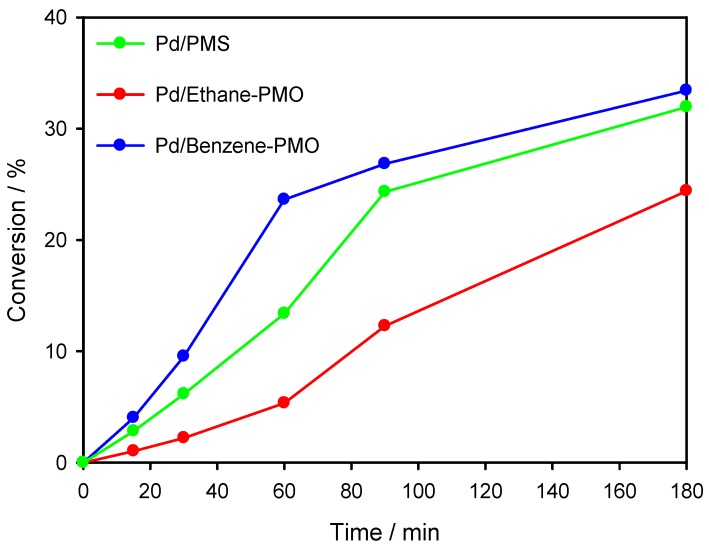
Overall conversion (mol%) in the Suzuki coupling of bromobenzene with phenylboronic acid on Pd-supported mesoporous materials.

**Figure 6:**
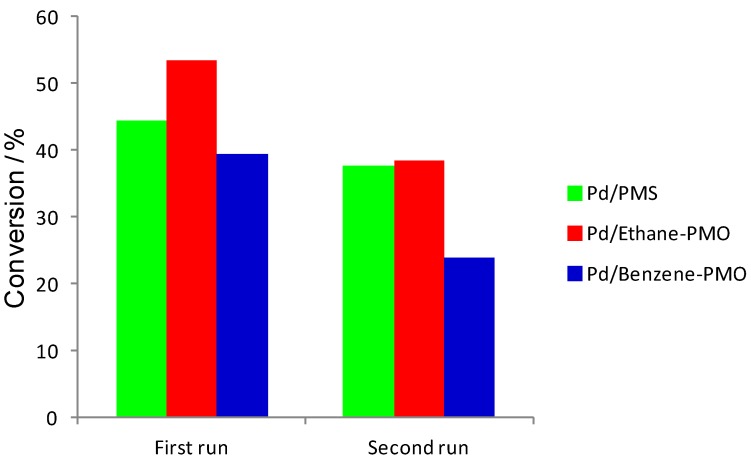
Overall conversion at 24 h in the Suzuki coupling of bromobenzene with phenylboronic acid on Pd-supported mesoporous materials in two consecutive runs.

The three Pd-supported mesoporous materials were tested as catalysts for the Suzuki cross-coupling reaction between bromobenzene and phenylboronic acid (Scheme 1). Preliminary tests allowed selecting K_2_CO_3_ as a convenient base. As shown in Figure 5, the three catalysts were active in the reaction at a relatively low reaction temperature (55 °C). The initial activity increased in the order Pd/Ethane-PMO < Pd/PMS < Pd/Benzene-PMO. However, higher reaction rates are generally observed with smaller Pd particles [20,21] and so other factors have to influence this reaction since the catalyst Pd/PMS exhibited the smaller average Pd particle size. Interestingly, the conversion at longer reaction times varied in a different way on the three catalysts (Figure 6). Thus, catalyst Pd/Ethane-PMO provided the highest conversion at 24 h, followed by Pd/PMS and finally by Pd/Benzene-PMO. Consequently, the activity of the latter catalyst hardly increased after 3 h. This fact could be related to the enhanced adsorption capacity of Benzene-PMO for aromatic compounds such as the reaction product (biphenyl) [7,22], thus causing a blockage of the metal centers in virtue of its unidimensional pore structure. Precisely, an increased adsorption for the reactants might explain the higher initial activity of this catalyst.

**Figure 7:**
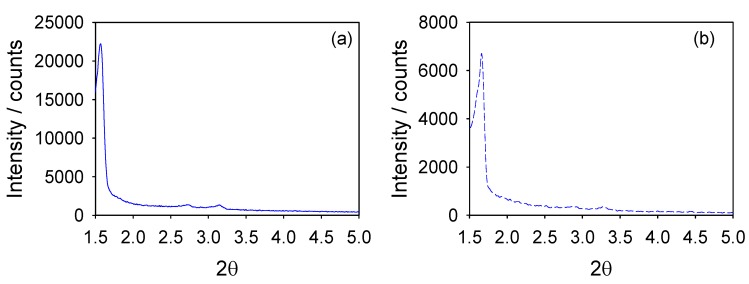
XRD patterns of the reduced sample Pd/Benzene-PMO (**a**) before; and (**b**) after being used as catalyst in the Suzuki cross-coupling reaction.

The three catalysts were evaluated for a second run after simple filtration (Figure 6). They were considerably deactivated, particularly those with organosilicas as supports. It has been reported that mesoporous materials can sometimes deactivate due to a progressive degradation of their mesostructure [23]. Accordingly, the stability of the mesoporous supports under the reaction conditions was studied. As can be observed in Figure 7, the mesostructure of all catalysts (only Pd/Benzene-PMO is shown) remained unaltered after the reaction. Although it is widely accepted that Pd-supported catalysts act as reservoirs of soluble Pd species [21,24], we believe that the significant deactivation observed on the catalysts upon reuse was mainly due to the deposition of organic and inorganic reagents and products on their surface, in agreement with other authors [21]. Currently, we are essaying reactivation procedures to recover the initial activity without damaging the mesoporous (organo)silica structure. Also, the synthesis of mesoporous materials with 3D structures would be certainly advantageous to address this issue [2,25,26].

## 3. Experimental Section

### 3.1. Synthesis of Catalysts

Periodic mesoporous silica, PMS, and organosilicas, *i.e.*, ethylene- and phenylene-bridged PMOs (Ethane-PMO and Benzene-PMO, respectively) were synthesized by using Brij-76 as surfactant under acidic conditions according to previously reported procedures [13]. The Si:Brij-76:HCl:H_2_O molar ratio in the mixture was 1:0.10:2.86:198. Surfactant-free materials obtained by three consecutive extractions with an acidic ethanolic solution were dried at 120 °C under vacuum for 10 h.

Palladium was deposited onto the mesoporous materials by impregnation to incipient wetness; a volume of 5 mL of acetone containing 0.03 g of palladium(II) acetylacetonate, Pd(acac)_2_, was added to 1.0 g of support to obtain a metal load of 1.0 wt.% Pd. The resulting suspension was stirred in a rotavapor at 40 °C for 24 h to remove the solvent. The impregnated materials were dried at 100 °C under vacuum.

### 3.2. Characterization of Catalysts

X-ray powder diffraction (XRD) patterns were recorded on a Siemens D-5000 powder diffractometer with a Bragg-Brentano geometry *(i.e.*, focused beam geometry), specifically a theta-2theta configuration, since the X-ray tube is stationary, the sample moves by the angle theta and the detector simultaneously moves by the angle 2theta. The instrument was equipped with a goniometer and a computerized data logging DACO-MP. The radiation used was Cu-Kα line (λ = 1.54 A). The diffractometer employed a Ni filter and a graphite monochromator. The voltage used was 40 kV and the intensity was 30 mA. The speed of the goniometer was 0.2 °/min, the scans were performed over the 2θ range from 1 (or 1.5, depending on the sample) to 9° and the measurement time was 40 min (or 37.5 min). The sample, previously dried and pulverized, was placed on a specimen holder so that the exposed surface was as flat and uniform as possible.

N_2_ isotherms were determined on a Micromeritics ASAP 2010 analyzer at −196 °C. The specific surface area of each solid was determined using the BET method over a relative pressure (P/P_0_) range of 0.05–0.30. Prior to measurement, the samples were outgassed at 120 °C for 24 h. The pore size distribution was obtained by analysis of the adsorption branch of the isotherms using the Barrett–Joyner–Halenda (BJH) method.

Temperature-programmed reduction (TPR) tests were performed on an AutoChem-2910 instrument equipped with a thermal conductivity detector (TCD), using a N_2_ stream containing 10% H_2_ at a flow-rate of 50 mL min^−1^ and a temperature ramp of 10 °C min^−1^ from –20 °C. The impregnated samples were previously outgassed in a He stream (35 mL min^−1^) at 100 °C for 1 h.

Hydrogen chemisorption experiments were carried out in a Micromeritics ASAP 2000 at 35 °C between 0 and 100 torr. Prior to measurement, the impregnated materials were reduced under H_2_ at 200 °C and subsequently degassed for 4 h at 180 °C and 10^−6^ mbar. A spherical model and a H/Pd adsorption stoichiometry of 1 were assumed to calculate metal dispersion and metal surface area.

Transmission electron microscopy micrographs (TEM) were taken using a PHILIPS CM-10 instrument, working at 80 kV and using copper grids. Only the samples reduced under the above-described conditions were studied by TEM.

### 3.3. Catalytic Activity

All reactions were conducted in a flask that was fitted with a reflux condenser and filled with a mixture of 1.0 mmol of bromobenzene, 1.5 mmol of phenylboronic acid and 5 mL of toluene. Following addition of the catalyst (0.11 g, 0.01 mmol Pd) and 2.0 mmol of K_2_CO_3_, the mixture was stirred at 55 °C. Prior to the reactions, the impregnated materials were reduced in an oven using a H_2_ stream at a flow-rate of 20 mL min^−1^ at 200 °C for 1 h.

Samples were analyzed in a Varian GC 3900 gas chromatograph (Agilent Technologies, Santa Clara, CA, USA), using a FactorFour VF-5ht capillary column (30 m × 0.25 mm ID), provided by Agilent Technologies (California), and raising the temperature from 50 to 200 °C at 20 °C min^−1^. Reaction products were identified by use of an online Varian Saturn 2100T mass spectrometer (Agilent Technologies, Santa Clara, CA, USA) and standards, and quantified with dodecane as internal standard.

## 4. Conclusions

Two periodic mesoporous organosilicas bearing either ethylene or phenylene bridges have been used as supports for Pd nanoparticles using an incipient wetness impregnation with Pd(acac)_2_ and subsequent reduction with hydrogen. Pd^2+^ species were more reducible in both organosilicas than in an analogous silica. Also, the silica support led to smaller particles, most of them located inside the pores, and consequently to a higher metal surface area and dispersion. The decisive role of the aromatic rings was evidenced by comparing both organosilicas. Thus, Pd nanoparticles supported on the material with ethylene bridges were spherical and mainly present on the external surface whereas those supported on the phenylene-bridged organosilica were cylindrical and confined in the mesopore structure. In addition, the latter material favored the reducibility of the Pd^2+^ species. All these Pd-supported materials were active in the Suzuki cross-coupling reaction between bromobenzene and phenylboronic acid. Although the Pd-supported phenylene-bridged organosilica provided the highest initial rate, the ethylene-bridged material showed the best performance at longer reaction times. However, all catalysts deactivated upon reuse in a different extension even though they were stable under the reaction conditions. Hydrophobic and, particularly, aromatic interactions seemed to greatly influence both the interaction between Pd^2+^ species and support as well as the adsorption of reactants and products on these materials.

Further investigations are being conducted in order to take advantage of the singular characteristics of Pd-supported periodic mesoporous organosilicas, particularly those containing phenylene bridges, and elucidate the role of the adsorption phenomena in their activity as well as in their deactivation and regeneration.
